# Whole genome sequence and de novo assembly revealed genomic architecture of Indian Mithun (*Bos frontalis*)

**DOI:** 10.1186/s12864-019-5980-y

**Published:** 2019-07-29

**Authors:** Sabyasachi Mukherjee, Zexi Cai, Anupama Mukherjee, Imsusosang Longkumer, Moonmoon Mech, Kezhavituo Vupru, Kobu Khate, Chandan Rajkhowa, Abhijit Mitra, Bernt Guldbrandtsen, Mogens Sandø Lund, Goutam Sahana

**Affiliations:** 1 Animal Genetics and Breeding Lab., ICAR-National Research Centre on Mithun, Medziphema, Nagaland 797106 India; 2 Center for Quantitative Genetics and Genomics, Department of Molecular Biology and Genetics, Aarhus University, 8830 Tjele, Denmark; 3 Present address: Dairy Cattle Breeding Division, ICAR-National Dairy Research Institute, Karnal, Haryana 132001 India

**Keywords:** Mithun, *Bos frontalis*, Genome, de novo assembly

## Abstract

**Background:**

Mithun (*Bos frontalis*), also called gayal, is an endangered bovine species, under the tribe bovini with 2n = 58 XX chromosome complements and reared under the tropical rain forests region of India, China, Myanmar, Bhutan and Bangladesh. However, the origin of this species is still disputed and information on its genomic architecture is scanty so far. We trust that availability of its whole genome sequence data and assembly will greatly solve this problem and help to generate many information including phylogenetic status of mithun. Recently, the first genome assembly of gayal, mithun of Chinese origin, was published. However, an improved reference genome assembly would still benefit in understanding genetic variation in mithun populations reared under diverse geographical locations and for building a superior consensus assembly. We, therefore, performed deep sequencing of the genome of an adult female mithun from India, assembled and annotated its genome and performed extensive bioinformatic analyses to produce a superior de novo genome assembly of mithun.

**Results:**

We generated ≈300 Gigabyte (Gb) raw reads from whole-genome deep sequencing platforms and assembled the sequence data using a hybrid assembly strategy to create a high quality de novo assembly of mithun with 96% recovered as per BUSCO analysis. The final genome assembly has a total length of 3.0 Gb, contains 5,015 scaffolds with an N50 value of 1 Mb. Repeat sequences constitute around 43.66% of the assembly. The genomic alignments between mithun to cattle showed that their genomes, as expected, are highly conserved. Gene annotation identified 28,044 protein-coding genes presented in mithun genome. The gene orthologous groups of mithun showed a high degree of similarity in comparison with other species, while fewer mithun specific coding sequences were found compared to those in cattle.

**Conclusion:**

Here we presented the first de novo draft genome assembly of Indian mithun having better coverage, less fragmented, better annotated, and constitutes a reasonably complete assembly compared to the previously published gayal genome. This comprehensive assembly unravelled the genomic architecture of mithun to a great extent and will provide a reference genome assembly to research community to elucidate the evolutionary history of mithun across its distinct geographical locations.

**Electronic supplementary material:**

The online version of this article (10.1186/s12864-019-5980-y) contains supplementary material, which is available to authorized users.

## Background

Mithun (*Bos frontalis*) is a rare bovine species living under free-range conditions inside tropical rainforest ecosystems of India, Bangladesh, Bhutan, China, and Myanmar [1]. It is a unique animal having a massive body, with characteristic ‘white stockings’ on their stout legs. This animal efficiently converts grass, forage, tree leaves as well as various agricultural by-products into highly nutritious meat. Moreover, mithun holds a unique place in the evolution of bovines. Mithun, having a specific chromosomal pattern, 2n = 58 is distinguishable from that of cattle (2n = 60) and yak (2n = 60) [2]. However, the origin of mithun is an on-going debate with no well-supported conclusion [3–5]. The deviation of the karyotype maybe originated from a 2/27 centric fusion or a Robertsonian translocation of cattle chromosomes 2 and 28 [6]. Besides ambiguity on its origin, information on genomic architecture of mithun is scanty so far.

Recently, researchers have carried out genomic studies on mithun. Mai et al. [7] reported whole-genome sequencing of mithun to detect single nucleotide polymorphisms (SNPs), copy number variations (CNVs), structural variations (SVs), SNP annotation and functional enrichment analysis of non-synonymous SNPs. Another research group presented the first genome assembly for gayal (mithun of Chinese origin) [8]. However, it is valuable to obtain genome sequence of individual from another geographical location to have better understanding on the genomic variation of mithun, which may also help to build a consensus assembly.

With rapid progress in sequencing technologies like next-generation sequencing (NGS) platforms, whole genomes of most livestock species have been sequenced to discover the underlying genetic architecture and explore species diversity, construct haplotype maps and perform genome-wide association studies. De novo assembly of many bovinae genomes including taurine cattle [9], indicine cattle [10], water buffalo [11], yak [12] and gayal [8] have been completed. Genome comparisons between closely related species provide insights into the genetic basis of mammalian divergence and adaptation [12]. Here we performed whole genome sequencing of one Indian adult female mithun (2n = 58,XX) using multiple sequencing platforms (Illumina HiSeq, Illumina Moleculo long reads and Pacific Biosciences: PacBio) [13–15] to generate a de novo genome assembly. This assembly was compared with genome assemblies of other species in the tribe *bovini* including the published gayal genome assembly [8]. We believe that an improved reference genome assembly would benefit understanding genetic divergence in mithun populations reared under diverse geographical locations and would be helpful in understanding the genomic architecture of this species.

## Results

### Genome assembly and assessment

**Fig. 1:**
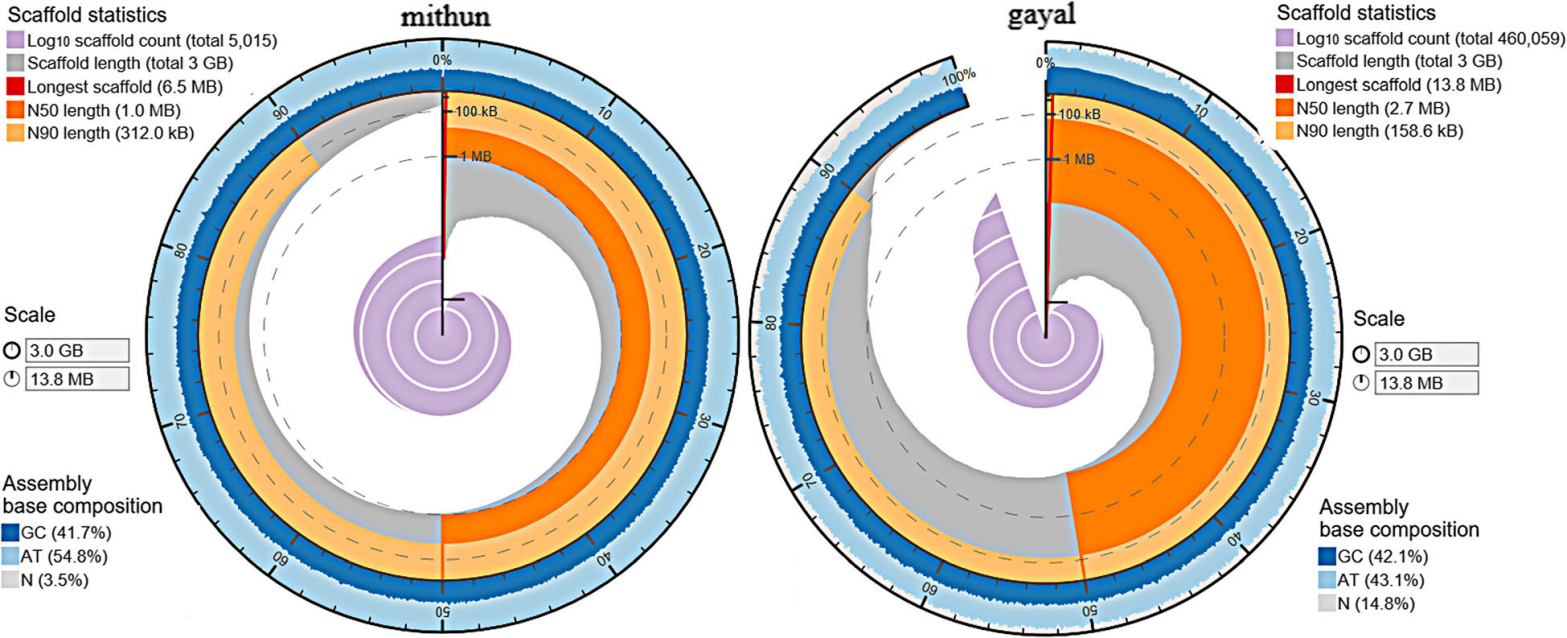
Assemblies statistics comparison between (**a**) mithun and (**b**) gayal [8]. The outside track showed the GC (dark blue), AT (light blue) and Ns (light gray). The middle track showed the length distribution of scaffolds with different categories: N90 length (earthy yellow), N50 length (orange) and all scaffolds (gray). The inner track showed the log10 of the scaffold counts (light purple)

Using multiple sequencing platforms, we generated a total of 241 Gb Illumina pair-end reads and mate-pair reads, 4.4 Gb Moleculo long reads and 4.8 Gb PacBio reads after removing adapter sequences and low quality reads. We adopted a hybrid strategy to assemble the genome. First, all the contaminating adapter sequences from the Illumina reads were removed, and then, the reads with low quality bases were trimmed. After that, paired-end reads were pre-assembled by an open access hybrid assembler MaSuRCA [16]. Next, these pre-assembled contigs were arranged in order and assembled into scaffolds by open-access script in SSPACE [17], combining with mate-pairs information. Finally, scaffolding was repeated by combining with Moleculo long reads and PacBio reads (corrected by LoRDEC [18]) by SSPACE-Long Read [19]. Genome size of mithun was estimated to be 3.09 Gb and 3.00 Gb was recovered in our assembly. A previous report estimated the genome size of gayal as 3.15 Gb and assembled the 2.85 Gb sequence with N50 value of 2.74 Mb [8]. Our genome assembly consists of 5,015 scaffolds. The size of the largest scaffold was 6,540,552 bp with N50 value as 1.00 Mb. The assembly of mithun genome presented here is less fragmented (5,015 vs. 460,059 scaffolds) and is more complete than the previously published gayal genome assembly [8] (Table 1, Fig. 1). Figure 1 showed that our mithun genome assembly was evenly distributed across the length of scaffolds. To assess the correctness of our assembly, we aligned paired-end reads and mate-pair reads onto the assembled mithun genome. The result showed 98.70% of the mate-pair reads could be aligned to our mithun genome assembly, 82.99% for 3 kb library and 84.42% of 5 kb library are properly paired with the mithun assembly (Table 2). This result proved a high degree of correct ordering and orientation of sequences in our mithun genome assembly. To check whether the genome included most of the protein coding genes, a BUSCO [20] analysis was performed. It was found that 91.50% of genes found in other *Bos* species were completely covered, while only 4.1% of genes were not present in the mithun assembly (Table 3). Compared with previous gayal assembly [8], our mithun assembly recovered more genes, which indicated our assembly has better coverage.

**Table 1:** Summary details of the mithun genome assembly compared with gayal genome

	Mithun genome	Gayal genome [8]
Total assembly length	3.00 Gb	2.85 Gb
Data Volume (after QC)	250 Gb	276 Gb
Contig count	480,463	583,373
Contig N50	11.5 Kb	14.4 Kb
Scaffold count	5,015	460,059
Largest scaffold	6.54 Mb	13 Mb
Scaffold N50	1.00 Mb	2.74 Mb
Gap count	301,766	2,647,378
Total gap length	106 Mb	421 Mb

**Table 2:** Sequence alignment of pair-end (PE) library, 3 Kb mate-pair (MP) library and 5 Kb MP library to assembly

	PE	3 Kb	5 Kb
Reads aligned	96.24%	98.71%	98.70%
Singletons	0.26%	0.87%	0.92%
Properly paired	90.81%	82.99%	84.42%
Mapped to different scaffolds	0.05%	13.86%	14.34%

**Table 3:** BUSCO gene completeness assessment

	Mithun	*Bos taurus* [21]	*Bos indicus* [22]	*Bos grunniens* [12]	Gayal [8]
Complete	91.5%	92.3%	90.1%	93.6%	85.2%
Fragments	4.4%	3.8%	3.8%	3.4%	7.8%
Missing	4.1%	3.9%	6.1%	3.0%	7.0%

### Repeat annotation

**Fig. 2:**
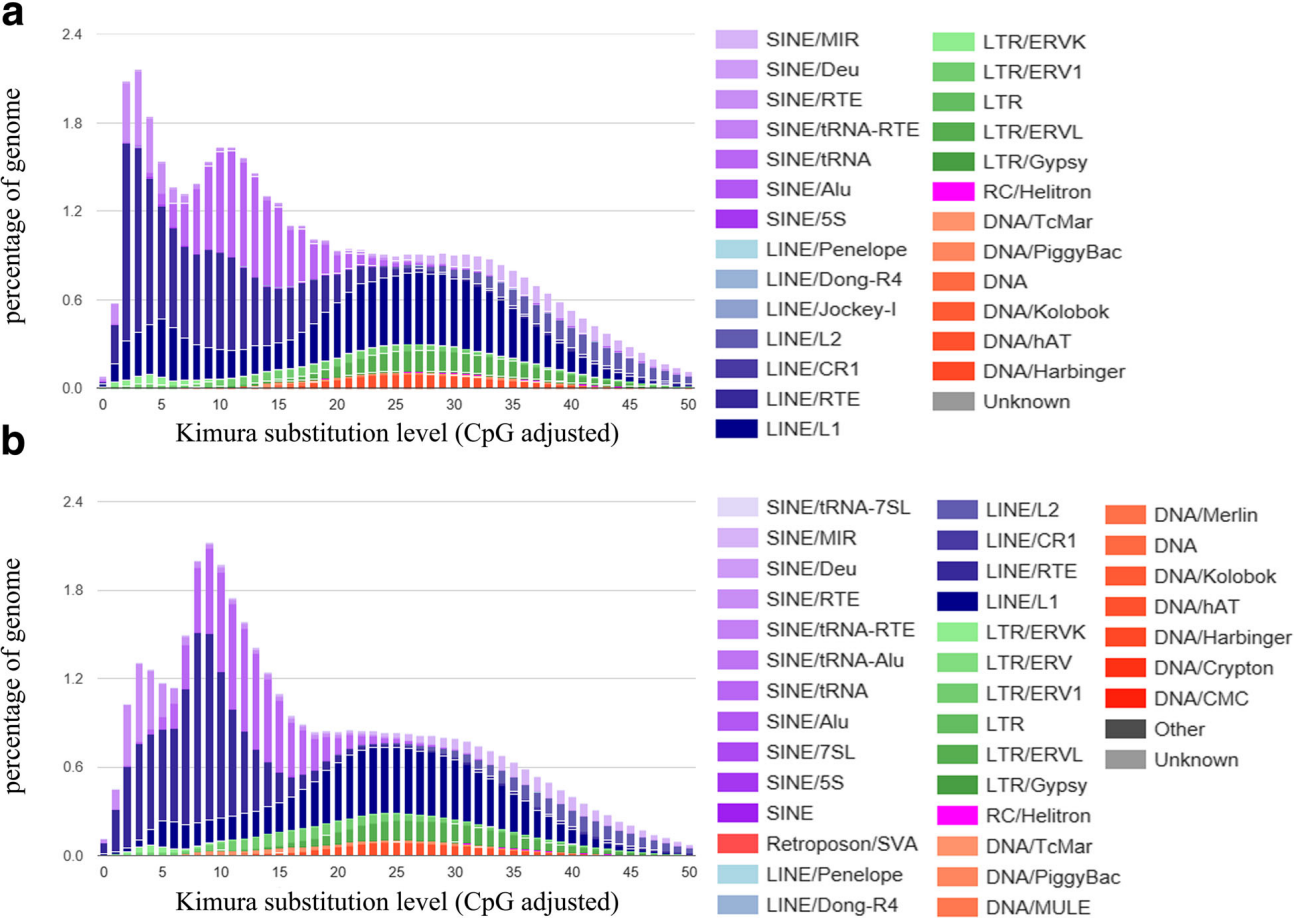
Interspersed Repeat Landscape of (**a**) cattle and (**b**) mithun. The y-axil shows the genome percent of each repeat families, the x-axil shows the level of kimura substitution of each repeat families

We applied the RepeatMasker program [23] using the mammalian repeat database [24] to screen DNA sequences for repeats. The result showed the mithun genome harbors 43.66% of repeat sequences, comparable to 49.38% in cattle genome (pre-analysis genome from http://www.repeatmasker.org/) and the previous gayal assembly (48.13%) [8]. The most abundant family was Long Interspersed Nuclear Elements (LINEs), followed by Short Interspersed Nuclear Elements (SINEs), which are common in mammalian genomes. Details of the genome proportion in each families are presented in Table 4. The substitution level of repeat sequences was estimated in mithun and compared with the cattle genome. As shown in Fig. 2, two genomes have similar pattern in the old repeat copies (number of substitutions greater than 20; more the substitutions, greater the age of the copy of the element). In contrast, differences were observed in more recently inserted elements with fewer substitutions compared to the consensus. Mithun genome has a high peak between 5 and 15 substitutions and a smaller peak with between 0 and 5. In contrast, most cattle repeat elements had between 0 and 5, and a small peak between 5 and 15 (Fig. 2). These differences originate from LINEs and SINEs in the genome.

**Table 4:** The repeat sequence composition in the mithun genome

Family	Percent of genome	Copy number of elements
LINEs	23.48	1,559,559
SINEs	11.84	2,413,359
LTR elements	4.69	438,811
Transposon	2.17	313,666
Small RNA	1.60	282,842
Satellites	1.29	89,091
Simple repeats	0.73	549,262
Low complexity	0.14	88,687
Unclassified	0.02	3,858
Total	43.66

### Gene annotation

**Fig. 3:**
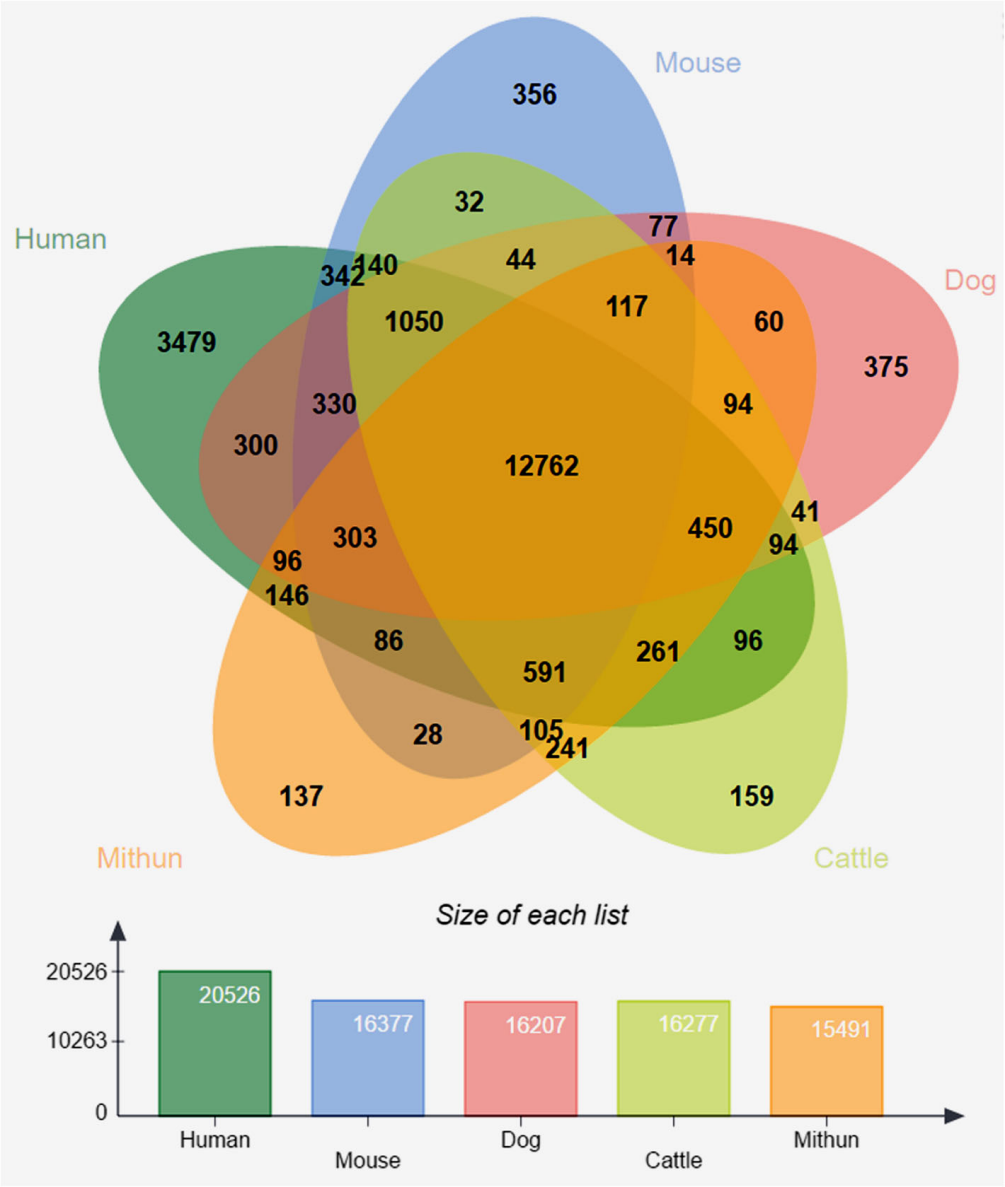
The orthologous groups and evolution of mithun genes. The distinct and shared ortholog groups of mithun, mouse, dog, human and cattle detected by OrthoMCL

Homology search, ab initio gene finding and transcriptome assembly were used to identify protein-coding genes. We identified 28,044 protein-coding genes in the mithun genome. Protein coding genes of orthologous groups were assigned by evolutionary genealogy of genes utilizing Non-supervised Orthologous Groups (eggNOG) mapper service [25], a public resource. We assigned 24,755 mithun genes to 15,491 orthologous groups. The orthologous groups of mithun were compared with those in human, mouse, dog and cattle genome (Fig. 3). As expected, several orthologous groups were only shared between cattle and mithun (241 in total). This number was much higher than those with other species (mithun vs. human: 146, mithun vs. mouse: 28, mithun vs. dog: 60). The domain of each protein coding sequence was scanned by InterProScan, an integration platform for the signature-recognition methods in InterPro [26], and 26,884 of 28,044 protein coding sequences were found to have at least one domain hit (Additional file 2: Table S1). The eggNOG mapper service [25] assigned possible gene names and the Gene Ontology (GO) [27] entries. A total of 26,041 sequences had hits in the eggNOG database. Among these, 22,107 had GO entries (Additional file 3: Table S2). We performed a Kyoto Encyclopedia of Genes and Genomes (KEGG) pathway [28] analysis by KEGG Automatic Annotation Server (KAAS) [29]. KEGG entries could be assigned to 11,725 genes (Additional file 4: Table S3).

### Genome alignment

**Fig. 4:**
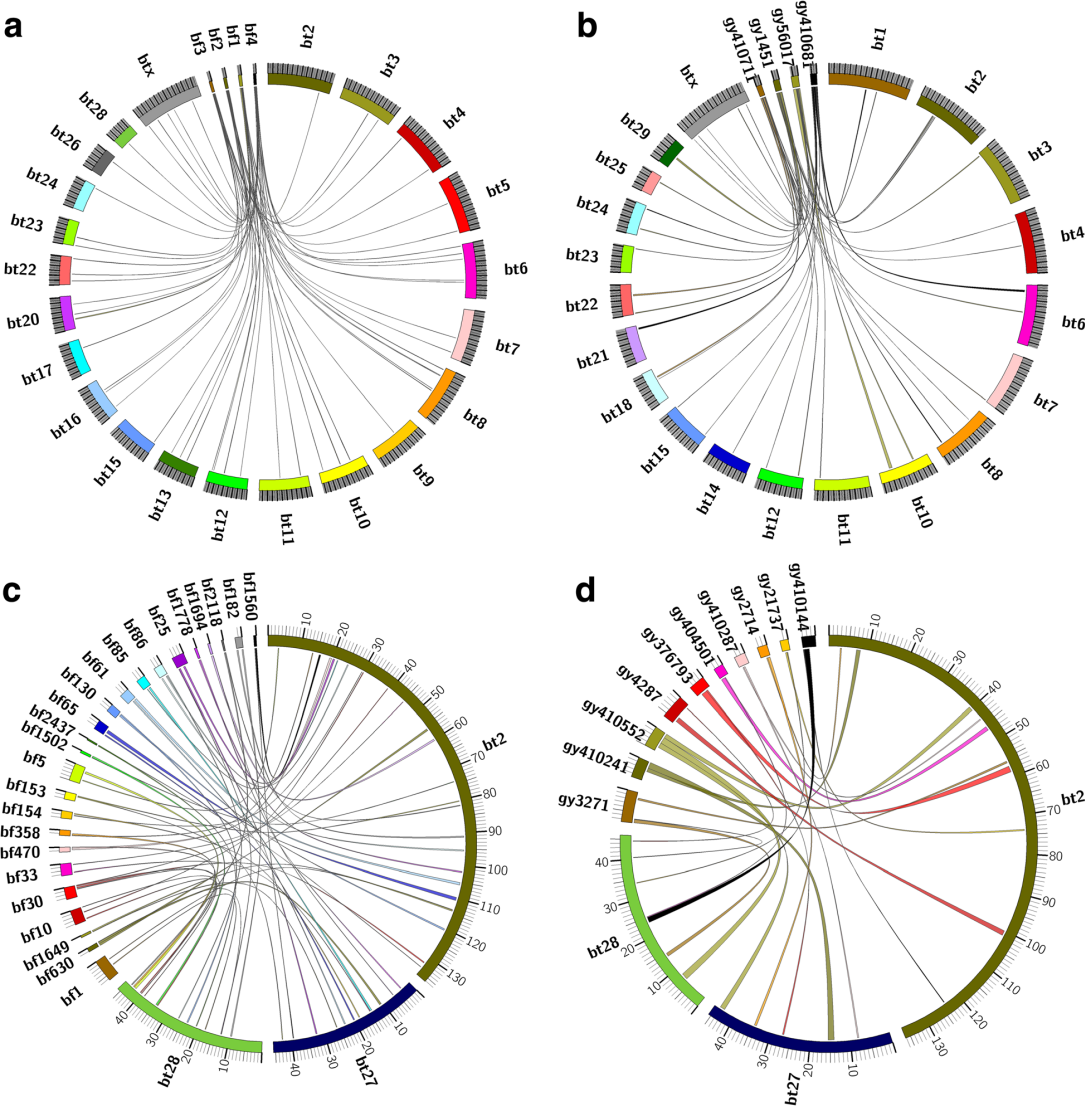
The genome alignment of mithun genome to cattle genome. The name of cattle chromosome have “bt” as prefix; mithun scaffold have “bf” as prefix. (**a**) The four longest scaffold of mithun and (**b**) gayal were drawn. The links in ring represent alignments which longer than 100 kb. (**c**) Mithun scaffolds and (**d**) gayal scaffolds which can span both cattle chromosome 2 and chromosome 27 or cattle chromosome 2 and chromosome 28 were drawn. The links in ring represent alignments which is longer than 100 Kb

Chromosome rearrangements between the mithun/gayal and cattle genomes were detected by aligning the mithun assembly to the cattle genome. 2.60 Gb of the 2.66 Gb cattle genome (leaving out Mt., Y chromosomes and unassigned sequences) aligned with the mithun sequence (98% alignment). We also aligned previously published gayal assembly [8] to cattle genome, and 2.62 Gb of the 2.66 Gb cattle genome could be covered (98%). As reflected by the N50 (Table 1) and scaffold size distribution (Fig. 1), the majority (6,288 of 15,089) of alignment blocks of our mithun assembly were having most synteny blocks ranging between 100 Kb ~ 1 Mb. In total, they covered 1.87 Gb of sequence (Table 5). In gayal assembly [8], there are more synteny blocks belong to 1 Mb to 10 Mb block size. However, the gayal assembly have more small synteny blocks compared with mithun assembly. As shown in Fig. 4a, the four longest scaffolds aligned with many cattle chromosomes (alignments longer than 100 Kb) and some of these scaffolds aligned to two chromosomes. The four longest scaffolds of gayal also had the similar alignment pattern as mithun (Fig. 4b). We also checked the genome alignment with cattle chromosomes 2, 27 and 28 to find any relic of chromosome fusion (Fig. 4c and d). If one of the mithun chromosome is fusion of cattle chromosome 2 and 27 or chromosome 2 and 28, we should see a scaffold span the fusion site from two cattle chromosomes. However, we did not find clear evidence to support this hypothesis due to the fragmentation of both assembly.

**Table 5:** The size distribution of synteny blocks of mithun genome aligned to cattle genome

Block size	Mithun		Gayal	
	Count of each block size	Total length for each block size (bp)	Count of each block size	Total length for each block size (bp)
10 bp–100 bp	718	43,780	762	48,762
100 bp-1 kb	2,336	925,165	6,419	2,688,643
1 kb–10 kb	1,673	6,227,751	3,940	11,741,636
10 kb–100 kb	3,918	204,420,988	1,387	57,277,708
100 kb-1 Mb	6,288	1,871,977,195	2,373	1,007,062,584
1 Mb–10 Mb	156	201,022,481	735	1,290,748,393
Total		2,588,165,483	2,616,185,027

## Discussion

### Motivation for mithun genome assembly

Mithun lives under free-range conditions in the tropical rain forests of the North Eastern Hill region of India, at an altitude ranging from 300 to 3,000 m above mean sea level. Mithun have great socio-economic importance among the mostly tribal population of the region. Mithun is primarily reared for meat, without any human inputs except occasional salt offerings [30]. Meat and milk of mithun have high quality in terms of higher fat% in milk and better marbling of its meat compared to cattle [31]. Besides, mithun may be resistant to mad cow disease [32]. These traits make mithun interesting for livestock research and for breeding. Even if the genome assembly of one gayal (mithun of Chinese origin) was recently published [8], the Indian mithun we sequenced here is from a diverse geographical location and separated for long time from the gayal animal sequenced previously [8]. Hence, we expected a high genome divergence between these two animals of different geographical origin. A superior genome assembly of mithun (Indian origin) will provide valuable information for research into mithun biology and genetics.

### Genome of Indian mithun

Here we presented the first de novo genome assembly of Indian mithun, which is more complete, less fragmented and better annotated (96%). By combining several next generation sequencing technologies, including some which generate long reads, we can avoid biases inherent in the individual technologies [33]. We used a hybrid genome assembly approach combining second and third generation sequencing data (combining short pair-end Illumina reads and Moleculo & PacBio long sequence reads) to produce an assembly with better genome coverage, fewer gaps and better scaffold statistics. It was also reported that performance of genome assembly improves significantly from hybrid approach using both short and long sequence reads [34]. Simultaneously, this approach also keeps overall costs of the projects manageable [35–39]. Some genomes viz. human [37], Korean chicken [40], gray mouse lemur [39], gorilla [41] and *Vibrio cholera* [42] have previously been assembled using similar strategies, where PacBio long reads helped to assemble genome regions rich in repetitive elements. Follow up research showed that single molecular sequencing could reduce assembly complexity of microbial genome [43]. Even for large genomes like human, the hybrid strategy can markedly improve its contiguity [44]. We assembled the genome into 5,015 scaffolds, which is less fragmented and has more complete genome coverage than the previously published gayal assembly [8] (Table 1).

A number of processes viz. annotation of genome, combined homology search, *ab into* prediction and a mithun transcriptome assembly [45] were used to identify protein-coding genes in the mithun genome. Genome annotation identified a high-quality set of 15,491 orthologous groups, a little less than cattle with about 16,000 groups. The number of gene orthologous families was lower than in other mammalian species (Fig. 2). Two primary reasons could account for this. Firstly, unlike chromosome-size scaffolds in cattle, the mithun genome assembly had smaller scaffolds. Because of this, some genes might be broken into two scaffolds and could not be detected. Secondly, we only had access to expression data from muscle tissue of mithun; therefore, we might have missed genes that were only expressed in other tissues or at different developmental stages. Nevertheless, our results greatly expand the information available on the gene sets present in the mithun genome.

### The origin and evolution of mithun

Despite years of cytogenetic and phylogenetic studies, no consensus has been reached on the origin of mithun. Mithun was first classified as an independent species in 1968 [46]. This conclusion was recently supported by the *cytochrome b* gene partial sequence [47]. Using mitochondrial DNA [48] and Y-chromosomal genes [46, 49], phylogenetic tree was constructed which showed a close relationship between the mithun and the gaur (*Bos gaurus*). This group was in turn found to be related to the banteng (*Bos javanicus*). Molecular phylogeny inferred from *cytochrome b* (Cytb), subunit II of *cytochrome c oxidase* (CO2), and the promotor of the *lactoferrin* gene (Lf) clustered mithun and banteng into one clade [50]. The gaur was not included in that study. Based on cytogenetic evidence, mithun [51] and gaur [50, 52] were reported to share a Robertsonian translocation involving the homologs of cattle chromosomes 2 and 28 when compared to cattle. Another study suggested that compared with cattle, mithun have a species-specific 2/27 centric fusion reducing the 60 chromosomes found in cattle to 58 chromosomes [6]. Neither of these conclusions was supported by our findings. To elucidate the origin of this unique species, either the chromosome level assembly of mithun (gayal) or sequencing mitochondrial genome of mithun taking a larger data set might be useful.

Patterns of genome-wide interspersed repeats in mithun showed both similarities to and differences from the pattern observed in cattle. The types of families observed in mithun and cattle were very similar. However, the distribution of the age of certain classes of repeat sequences clearly differed. Sequence divergence among LINEs and SINEs peaked at a much higher value in mithun than in cattle. This reflects that the numbers of LINEs and SINEs in mithun have expanded a relatively long time ago. In contrast, expansion of LINE and SINE numbers must have been much more recent in cattle. During the course of this study, we could see that the number of ancient repeat elements were very similar in these two species, but cattle have more repeat elements those are recently active (big peak of substitution level below 5 for cattle). Therefore, these two species have different evolution pattern after they diverged.

## Conclusion

The main objective of the present study is to furnish a genetic resource and a de novo reference genome of mithun to facilitate future research. Our de novo draft assembly is the first genome assembly of Indian mithun, which is constructed using a hybrid approach. This improved the overall performance of the genome assembly. Our assembly is less fragmented, having better coverage and is completed to a reasonable extent. We believe this mithun genome assembly will provide genomic resource to evolutionary studies in combination with other bovine species, and will help to understand the genomic architecture of various phenotype and genotype interactions underlying this unique bovine species from distinct geographical habitat.

## Methods

For sequencing of genomic DNA, blood sample was collected by a qualified Veterinarian in vacuutainer tube containing EDTA (Becton Dikinson, USA), from the jugular vein of one healthy adult female mithun (2n = 58,XX), maintained in the Institute research farm, Medziphema, Nagaland, India under semi-intensive rearing system. The standard animal ethics normswere followed and care of the animal was taken in accordance with guidelines of the Committee for the Purpose of Control and Supervision on Experiments on Animals (CPCSEA), prescribed by the Indian Council of Agricultural Research (ICAR), Ministry of Agriculture and Farmers Welfare, Government of India. In an earlier study on gene expression, the muscle samples were collected for RNA extraction from growing male mithuns, average 24 months of age (range 19–29 months) under standard anaesthesia by a qualified Veterinary Surgeon, from the Institute Research Farm, Medziphema. Institutional Animal Ethics Committee had approved collection of mithun blood and muscle samples for the purpose of DNA and RNA extraction. All these procedures under the present study agrees with the ARRIVE Guidelines for reporting research [53] involving animals (Additional file 1). 

### DNA isolation, libraries preparation and sequencing

#### Paired-end sequencing

Genomic DNA from blood was prepared using QIAamp DNA Mini Kit (Qiagen) and was quantified using Qubit DNA BR Quantitation kit (Invitrogen). The genomic library was prepared according to the manufacturer’s protocol (Illumina, True Seq DNA preparation guide) using the Illumina TruSeq DNA LT library kit. The paired-end library was sequenced on an Illumina HiSeq 2500 in 2 × 100 cycles using the SBS sequencing kits V3.0, generating a total of 201.06 Gb of paired-end data. These sequence data was submitted in NCBI Database (BioProject ID PRJNA241403).

#### Mate-pair sequencing

Following fragmentation, the DNA fragments were end-repaired with labeled dNTPs. The DNA fragments were circularized, and non-circularized DNA was removed by digestion. Circular DNA was fragmented, and the labeled fragments (corresponding to the ends of the original DNA ligated together) were purified using affinity chromatography. Purified fragments were end-repaired and ligated to Illumina paired-end sequencing adapters. Additional sequences complementary to the flow cell oligonucleotides were added to the adapter sequence with tailed PCR primers. The final libraries prepared in this process were consisted of short fragments made up of two DNA segments, originally separated by several kilobases. Two separate mate-pair libraries of 3 kb each and three libraries of 5 kb each were prepared using Illumina Nextera Mate-Pairs sample preparation kit as per manufacturer’s protocol. These libraries were then sequenced using Illumina NGS platform (HiSeq 2500) to generate a total of 40.40 Gb high quality and cleaned mate-pair sequence reads.

#### Illumina moleculo long-reads

Illumina TruSeq synthetic long-read technology was used to generate moleculo long reads in this study. The protocol involves initial mechanical fragmentation of genomic DNA into 10 kb fragments. These fragments then undergo end-repair and ligation of amplification adapters, before diluted onto 384-well plates so that each well contains DNA representing approximately 1–2% of the genome (200 molecules, in the case of *D. melanogaster*). Polymerase chain reaction (PCR) was used to amplify molecules within wells, followed by parallel Nextera-based fragmentation and barcoding of individual wells. DNA from all wells was then pooled and sequenced on the Illumina HiSeq 2000 platform. Data from individual wells were demultiplexed*in silico* according by barcode sequences, generating approx. 4.4 Gb clean sequence data. Synthetic long-reads were assembled from the short reads using a specific assembly pipeline.

#### PacBio sequencing

PacBio (Pacific BioSciences) long read sequencing technique, enabled by the SMRTbell® (Single-molecule Real Time) technology was employed in this study. The SMRTbell® template preparation method creates a circularized template for use with multiple sequencing protocols. A single streamlined protocol was used to create different insert size libraries i.e. 10 kb and 20 kb by altering the fragmentation conditions. The first step in the generation of a SMRTbell library was production of appropriately sized double-stranded DNA fragments. These fragments can be generated by random shearing of DNA, or by amplification of target regions of interest. The SMRTbell library was produced by ligating universal hairpin adapters onto double-stranded DNA fragments. The hairpin dimers formed during this process were removed at the end of the protocol using a magnetic bead purification step with size-selective conditions. The final step of the protocol was to remove failed ligation products with exonucleases. After the exonuclease step, SMRTbell templates were annealed to primers, and annealed templates were bound to DNA polymerase. Lastly, the sample plate was set up for sequencing.

### RNA extraction, cDNA synthesis, library preparation and sequencing

RNA was extracted from each of the four muscle tissues following standard guidelines of Illumina Low Sample Protocol (TruSeq® RNA Sample Preparation v2 Guide). In brief, total RNA integrity following isolation was checked using an Agilent Technologies 2100 Bioanalyzer for each sample with an RNA Integrity Number (RIN) value greater than or equal to eight. The first step in the workflow involved purifying the poly-A containing mRNA molecules using poly-T oligo-attached magnetic beads. Following purification, the mRNA was fragmented into small pieces using divalent cations under elevated temperature. The cleaved RNA fragments were copied into first strand cDNA using reverse transcriptase and random primers. This was followed by second strand cDNA synthesis using DNA Polymerase I and RNase H. These cDNA fragments then went through an end repair process, the addition of a single ‘A’ base, and then ligation of the adapters. The products were then purified and enriched with PCR to create the final cDNA library. This protocol for transcriptome analysis was performed on RNA after mRNA purification using elevated temperatures, resulting in libraries with insert size ranging from 120 to 200 bp with a median size of 150 bp. Transcriptome sequencing was carried out using the Illumina Hi-seq 2000 platform to generate paired-end reads. The RNAseq data are deposited in the NCBI Database (BioProject accessions: PRJNA307305; BioSample accessions: SAMN04384021, SAMN04384020, SAMN04384019 and SAMN04384018).

### Genomic data processing, genome assemble and assembly assessment

Trimmomatic [54] was used to remove the adaptor and trim the raw data of Illumina paired-end (PE) sequencing and mate-pair (MP) sequencing data. High quality PE data were assembled by MaSuRCA [53]. The contigs obtained from MaSuRCA and cleaned MP data were scaffold by SSPACE [17]. PacBio data were error-corrected and trimmed by LoRDEC [55] by using error corrected PE data. Then we re-scaffolded the assembly by SSPACE-Long Read with error corrected PacBio data and raw illumine Moleculo Long reads. Thereafter, the assembly was polished by PBJelly 2 [56]. Final statistics of the assembly were assessed by QUAST [57]. Part of the PE and MP data were mapped to the draft genome by BWA [58]. Properly paired reads reported by samtools [59] flagstat were used to investigate the correctness of assembly. BUSCO v3 [20, 60] with the mammalian database was used to assess the completeness of genes presented by assembly. Nineteen-mers was counted from PE data with Jellyfish [61]. Genome size was estimated by dividing the total number of k-mers by the peak value of the k-mer frequency distribution [62].

### RNA-seq data processing

RNA-seq data having adaptor sequences were removed and low-quality bases (average quality per base drops below 15 in 4 bases sliding windows) were trimmed using Trimmomatic [54]. RNA-seq data from four samples were mixed together to help to build a comprehensive muscle transcriptome. The mixed data set were de novo assembled by Trinity [63]. We also performed genome guided assembly by following procedure: the mixed dataset were aligned to genome assembly by Tophat2 [64]. Trinity then assembled the aligned reads. We also generated the transcriptome by Cufflink [65].

### Repeat sequence annotation

The genome assembly were masked by RepeatMasker [23] with the mammalian database. The substitution level (alignment of each repeat element sequences with their consensus sequence in database) calculation and plots were done using *calcDivergenceFromAlign.pl* and *createRepeatLandscape.pl* scripts provided with RepeatMasker. The *RepeatLandscape* for cattle genome assembly (BTA7) was downloaded from the RepeatMasker website of pre-analysis species. The statistics for the BTA7 assembly was also downloaded from RepeatMasker website and compared with mithun.

### Gene annotation

We annotated the mithun genome by combination of three strategies: Ab Initio gene prediction, protein homology search and a transcriptome assembly. Homology search was scanned by Exonerate [66] against mammalian protein sequences collected from Uniport [67]. Trinity [63] de novo assembly, Trinity [63] genome-guided assembly and TopHat2 plus Cufflinks [64] assembly was merged to build a comprehensive transcriptome by PASA pipeline [68]. Ab initio gene prediction was performed by Augustus [69], using the configure file trained by BUSCO [20]. In addition, we provided the RepeatMasker [23], Exonerate [66], PASApipeline [68] and Tophat2 [64] alignment of RNA-seq data as hints for Augustus [69]. These three set of annotations were merged by EVM [70] by weighting them as ab initio gene prediction: 1; Homology search: 6 and transcriptome: 10 as suggested in the EVM manual [70]. For mithun reference genes, motifs and domains were detected by InterProScan [26] against multiple database including Pfam [71], Panther [72], PRINTS [73], Gene3D [74], SUPERFAMILY [75]. The GO terms of each gene was assigned by eggNOGmapper [25]. We also used KASS [76] to identify the KEGG [77] pathway information of [77] pathway information of the mithun gene set.

### Genome alignment

Both mithun and gayal genomes were soft-masked and aligned to the soft-masked cattle genome (ARS-UCD1.2) [21] by Large Scale Genome Alignment Tools (LASTZ) [78]. The pairwise genome alignment was chained according to their location in both genomes by axtChain program [79]. The netting process chooses for the reference species the best sub-chain in each region. The statistics of different size of synteny block was done by a custom script. We only used block size larger than 100 kb to investigate how many cattle chromosomes the mithun scaffold can span.

## Additional files

Additional file 1: NC3Rs ARRIVE Guidelines Checklist. (PDF 160 kb)

Additional file 2: **Table S1.** Genes found in mithun genome with at least one domain hit. (XLSX 2390 kb)

Additional file 3: **Table S2.** Genes found in mithun genome with GO analysis. (XLSX 5227 kb)

Additional file 4: **Table S3.** Genes found in mithun genome with KEGG entries. (XLSX 510 kb)

## Data Availability

All data generated and analyzed during this current study are available in the Data Cell of ICAR-NRC on Mithun, Nagaland, India with permission from the Competent Authority. Illumina whole genome sequence and RNA-seqdata of mithun were submitted in NCBI Database having BioProject ID PRJNA241403 and BioProject ID PRJNA307305, respectively.

## Abbreviations

BTA 7: *Bos taurus* genome assembly v.7
BUSCO: Benchmarking Universal Single-Copy Orthologs
CNVs: Copy number variations
DBG: De Bruijn Graphs
dNTPs: Deoxyribonucleotide triphosphates
eggNOG: Evolutionary genealogy of genes: Non-supervised Orthologous Groups
EVM: EVidence Modeler
Gb: Gigabyte
KAAS: KEGG Automatic Annotation Server
KEGG: Kyoto Encyclopedia of Genes and Genomes
LASTZ: Large-Scale Genome Alignment Tool
LINEs: Long Interspersed Nuclear Elements
LoRDEC: Long Read DBG Error Correction
LTR: Long terminal repeat
MaSuRCA: Maryland Super-Read Celera Assembler
MB: Megabyte
Mt.: Mitochondria
NGS: Next-generation sequencing
PacBio: Pacific BioSciences
PASApipeline: Program to Assemble Spliced Alignments Pipeline
PBJelly 2: Software for Long-Read Sequencing Data from PacBio
Pfam: Protein families database
PRINTS: Protein fingerprints database
QUAST: Quality Assessment Tools
RIN: RNA Integrity Number
SINEs: Short Interspersed Nuclear Elements
SMRT: Single-molecule real-time (SMRT)
SNPs: Single-Nucleotide Polymorphisms
SSPACE: Scaffolding Pre-Assemblies AfterContig Extension
SVs: Structural variations
